# Women With Diabetes Are at Increased Relative Risk of Heart Failure Compared to Men: Insights From UK Biobank

**DOI:** 10.3389/fcvm.2021.658726

**Published:** 2021-04-06

**Authors:** Sucharitha Chadalavada, Magnus T. Jensen, Nay Aung, Jackie Cooper, Karim Lekadir, Patricia B. Munroe, Steffen E. Petersen

**Affiliations:** ^1^William Harvey Research Institute, NIHR Barts Biomedical Research Centre, Queen Mary University of London, Charterhouse Square, London, United Kingdom; ^2^Barts Heart Centre, St Bartholomew's Hospital, Barts Health NHS Trust, West Smithfield, London, United Kingdom; ^3^Department of Cardiology, Copenhagen University Hospital Amager & Hvidovre, Hvidovre, Denmark; ^4^Artificial Intelligence in Medicine Lab (BCN-AIM), Departament de Matemàtiques and Informàtica, Universitat de Barcelona, Barcelona, Spain

**Keywords:** diabetes, heart failure, sex, prospective, UK biobank, epidemiology, cardiovascular, prognosis

## Abstract

**Aims:** To investigate the effect of diabetes on mortality and incident heart failure (HF) according to sex, in the low risk population of UK Biobank. To evaluate potential contributing factors for any differences seen in HF end-point.

**Methods:** The entire UK Biobank study population were included. Participants that withdrew consent or were diagnosed with diabetes after enrolment were excluded from the study. Univariate and multivariate cox regression models were used to assess endpoints of mortality and incident HF, with median follow-up periods of 9 years and 8 years respectively.

**Results:** A total of 493,167 participants were included, hereof 22,685 with diabetes (4.6%). Two thousand four hundred fifty four died and 1,223 were diagnosed or admitted with HF during the follow up periods of 9 and 8 years respectively. Overall, the mortality and HF risk were almost doubled in those with diabetes compared to those without diabetes (hazard ratio (HR) of 1.9 for both mortality and heart failure) in the UK Biobank population. Women with diabetes (both types) experience a 22% increased risk of HF compared to men (HR of 2.2 (95% CI: 1.9–2.5) vs. 1.8 (1.7–2.0) respectively). Women with type 1 diabetes (T1DM) were associated with 88% increased risk of HF compared to men (HR 4.7 (3.6–6.2) vs. 2.5 (2.0–3.0) respectively), while the risk of HF for type 2 diabetes (T2DM) was 17% higher in women compared to men (2.0 (1.7–2.3) vs. 1.7 (1.6–1.9) respectively). The increased risk of HF in women was independent of confounding factors. The findings were similar in a model with all-cause mortality as a competing risk. This interaction between sex, diabetes and outcome of HF is much more prominent for T1DM (*p* = 0.0001) than T2DM (*p* = 0.1).

**Conclusion:** Women with diabetes, particularly those with T1DM, experience a greater increase in risk of heart failure compared to men with diabetes, which cannot be explained by the increased prevalence of cardiac risk factors in this cohort.

## Introduction

Globally, more than 500 million people have diabetes and its prevalence (6–7% in the UK) is expected to increase (1). The risk of all-cause mortality is doubled in individuals with diabetes compared to those without diabetes, with cardiovascular disease being the leading cause of death (2, 3). Approximately £3billion of the £10billion total cost of diabetes to the National Health Service (NHS) is associated with the cardiovascular complications of diabetes, and this figure is projected to increase to almost double in the next 20 years (4). Accelerated heart failure (HF) is a common manifestation of cardiovascular disease in people with diabetes and can occur independently of macrovascular coronary disease (5–7). In fact, non-ischaemic cardiomyopathy is the earliest and most common cardiovascular complication in people with diabetes (8).

Diabetic cardiomyopathy was first described in the 1970s (9) and is referred to as a process that affects cardiac structure and function independent of age and cardiovascular risk factors, or events which can lead to diastolic or systolic heart failure. The European Society of Cardiology has recently recognized this as a special subset of heart disease (10).

A consistent pattern has emerged revealing a prominent sex difference in the risk of developing HF from diabetes. In a recent meta-analysis of 12 million individuals, the risk of HF related to type 1 diabetes was more than 5-fold higher in women but only 3.5 times higher in men compared to individuals without diabetes. Similarly the risk of HF related to type 2 diabetes was 9% higher in women than in men (11). While the meta-analysis showed a consistent pattern, the data included were heterogenous from multiple studies with unharmonized data and therefore did not allow for further exploration of relevant contributing factors.

Using UK Biobank, a prospective population cohort study, and its large-scale detailed individual participant information, we investigated the association between presence of diabetes, sex, and risk of heart failure. We hypothesized that the increased relative risk of heart failure associated with diabetes in women compared to men would persist despite accounting for detailed individual level characteristics. We also investigate mortality as an endpoint but it not the primary focus of this study.

## Methods

### Study Population

The UK Biobank was a prospective population study of half a million people aged 40–69 years when recruited between 2006 and 2010. The data collected, and summary of the characteristics can be viewed on the UK Biobank's website (www.biobank.ac.uk).

This study includes the entire UK Biobank cohort after excluding 30 participants who withdrew from the study before analysis of data (502,506). Participants diagnosed with Diabetes Mellitus (DM) after enrolment were also excluded from the study (*n* = 9,339). The UK Biobank population was stratified as our exposure into non-diabetes, type 1 diabetes, and type 2 diabetes by the method previously suggested (12).

### Ethics

This study complies with the Declaration of Helsinki. The study was covered by the ethical approval for UK Biobank studies from the National Health Service National Research Ethics Service on June 17, 2011 (Ref 11/NW/0382) and extended on May 10, 2016 (Ref 16/NW/0274) with informed consent obtained from all participants.

### Study Design

The start of the study was recorded as the date of attending the first assessment for the UK Biobank study. Age of diabetes diagnosis was recorded from self-reported data and where missing supplemented using Hospital Episode Statistics (HES) data. If the diagnosis of diabetes was made after the participant attended the first assessment for the study, then these participants were excluded from the study. Time dependent co-variate analysis was considered to include participants that developed diabetes after enrollment. However, ultimately abandoned as this is reported to introduce serious bias when used with competing risk analysis (13). Additionally, the results did not differ when using diabetes as time-dependent or fixed variable, thus further supporting use of diabetes as a fixed variable.

The endpoints of death and heart failure were derived from HES data with dates recorded to provide censor dates. All-cause mortality and incident heart failure, as our outcomes, were derived in the whole UK Biobank population.

We extracted possible confounders for the effect of diabetes on the outcomes all-cause mortality and incident heart failure. Co-morbidities including hypertension, and hypercholesterolaemia were defined using a combination of self-reported data and supplemented with the medication history (see Appendix A for further details). Defining those with coronary artery disease included self-reported data, HES data and included hospital admissions with angina as well as any coronary event or intervention. Coronary disease is considered to be an important confounder in this study; therefore, a robust definition was made to have a broad capture of individuals with any clinically significant coronary disease in order to reduce any residual confounding.

Sex, ethnicity, smoking and alcohol were recorded from self-reported data fields. Smoking and alcohol status were categorized into never, previous and current use status. The use of diabetic oral medication or insulin use was derived from the self-reported medication use field.

Body Mass Index (BMI) recorded from calculated BMI based on their first assessment of height and weight. Participants' level of physical activity was calculated using frequency (number of days/week) and duration (minutes/d) of walking, moderate intensity, and vigorous-intensity exercise. A continuous value for the amount of physical activity, measured in metabolic equivalent minutes/week (METs), was calculated by weighting different types of activity (walking, moderate, or vigorous) by its energy requirements using values derived from the IPAQ study (International Physical Activity Questionnaire)(14). This was then further categorized according to the World Health Organization recommendation for physical activity (15) and were subdivided into below recommendation, above recommendation or meets recommendation.

### Statistical Analysis

The UK Biobank population were first divided into those with and without diabetes. Those without diabetes were the reference group for comparison in all analyses. A univariate analysis was carried out to assess mortality differences in those with diabetes and those without diabetes. Kaplan-Meier analysis was used to demonstrate these results. The associations of DM, type of DM stratified by sex with endpoints were analyzed using a multivariate cox regression model. The covariates included in the model were age, BMI, smoking and alcohol status (current, previous, never or unknown), ethnicity, hypertension, hypercholesterolaemia and coronary disease. The proportional hazards assumption was tested for all models using the Schoenfeld residuals. The assumption was violated when prevalent coronary artery disease was included as a covariate and so a stratified model was fitted. The significance of the differential associations between diabetes and sex with outcomes were tested using an interaction term. The 95% confidence interval for the difference in coefficients for men vs. women in each model was obtained with bootstrapping (1,000 times). For heart failure a competing risk analysis was conducted using a Fine and Gray competing risks model (16) to assess any impact of informative censoring as those dying from other causes may also have higher heart failure risk. A sensitivity analysis was also performed where any participants with coronary disease were excluded. The competing risk analysis support the results from the multivariate cox models. We also performed secondary sensitivity and mediation analysis. All analyses were performed with R studio version 1.2 (17). The R packages used for statistical analysis include the “survival,” “boot,” and “regmedint” packages.

## Results

A total of 493,167 participants were included in this study. A total of 22,685 participants (4.6%) had prevalent diabetes at the start of the study. The population was further divided by sex with 260,743 (55%) female participants without diabetes and 8,531 (38%) female participants with diabetes. HF occurred in 6,137 participants (1,223 with DM and 4,914 without DM) and 19,590 participants died (2,454 with DM and 17,136 without DM). For heart failure, the median follow-up was 8 years (IQR 7–9 years) and for all-cause mortality, the median follow-up was 9 years (IQR 8–10 years).

### Baseline Characteristics

Overall, the type 2 diabetes sub-group was older with a higher BMI and higher cholesterol levels compared to the control group (Table 1). In contrast, the median BMI of those with type 1 diabetes was only marginally increased compared to those without diabetes and the average age was the same as for the control group without diabetes. The duration of diabetes was longer in type 1 diabetes compared to type 2 diabetes, which is expected since they are diagnosed at a younger age. The participants with diabetes had a lower proportion of participants that either met or exceeded the World Health Organization (WHO) recommended level of physical activity. As expected, diabetes was associated with a higher prevalence of coronary disease, diagnosed hypertension, and hyperlipidaemia.

**Table 1:** Participants' characteristics.

	**Control**	**Participants with diabetes**	***P*-value**	**Type 1 DM**		**Type 2 DM**		***P*-value**
Total, n	470,482	22, 685		2,626		20,059		
**Demographics**								
Age at enrolment (years), mean (sd)	56 (8.1)	60 (7.1)	<0.001	57 (8.2)		60 (6.9)		<0.001
Female sex, *n* (%)	260,743 (55%)	8,531 (38%)	<0.001	1,123 (43%)		7,408 (37%)		<0.001
Ethnicity, *n* (%)			<0.001					<0.001
Caucasian	444,873 (94.5%)	19,638 (87%)		2,395 (91.2%)		17,243 (85.9%)		
Afro-Caribbean	6,994 (1.5%)	752 (3.3%)		72 (2.8%)		680 (3.4%)		
South-Asian	6,405 (1.4%)	1,252 (5.5%)		74 (2.8%)		1,178 (5.9%)		
Other	12,210 (2.6%)	1,043(4.2%)		85 (3.2%)		958 (4.8%)		
**Lifestyle factors**								
Smoking *n* (%)			<0.001					<0.001
Never	258,631 (55%)	10, 189 (45%)		1,325 (50.5%)		8,864 (44.2%)		
Previous	159,907 (34%)	9,763 (43%)		940 (35.8%)		8,823 (44%)		
Current	49,462 (10.5%)	2,506 (11%)		343 (13%)		2,163 (10.8%)		
Unknown	2,482 (0.5%)	227 (1%)		18 (0.7%)		209 (1%)		
Alcohol *n* (%)			<0.001					0.12
Never	19,586 (4.2%)	2,031 (9%)		206 (7.8%)		1,825 (9.1%)		
Previous	15,780 (3.3%)	1,712 (7.5%)		202 (7.7%)		1,510 (7.5%)		
Current	433,683 (92.2%)	18,823 (83%)		2,208 (84.1%)		16,615 (82.8%)		
Unknown	1,433 (0.3%)	119 (0.5%)		10 (0.4%)		109 (0.6%)		
Physical activity – meeting or above WHO recommendatio*n* (%)	279,296 (59%)	10767 (47%)	<0.001	1,409 (54%)		9,444 (47%)		0.018
BMI, median, kg/m^2^, (IQR)	26.5 (24.0–29.6)	30.6 (27.3–34.7)	<0.001	27.4 (24.4–31.1)		31.0 (27.8–35.0)		<0.001
**Medical background**								
Duration of diabetes mellitus, median years, y, (IQR)	0 (0–0)	14 (11–19)	NA	Male: 28 (18–41)	Female: 27 (17–40)	Male: 14 (11–18)	Female: 13 (10–17)	<0.001
Hba1c (mmol/mol), median (IQR)	35 (33–37)	51 (44–60)	<0.001	Male: 59 (50–68)	Female: 61 (54–70)	Male: 51 (44–59)	Female: 50 (44–58)	<0.001
Diagnosed/treated for coronary artery disease	18,324 (3.9%)	3,947 (17.4%)	<0.001	400 (15.2%)		3,547 (17.7%)		0.002
Diagnosed/treated for hypertension	121,005 (25.7%)	15,709 (69.2%)	<0.001	1,496 (57%)		14,213 (70.9%)		<0.001
Diagnosed/treated for hyperlipidaemia	70,100 (14.9%)	14,789 (65.2%)	<0.001	1,469 (55.9%)		13,320 (66.4%)		<0.001

*WHO, World Health Organization; BMI, body mass index; IQR, interquartile range; Hba1c, glycated hemoglobin; SD, standard deviation*.

### Diabetes and All-Cause Mortality

**Figure 1:**
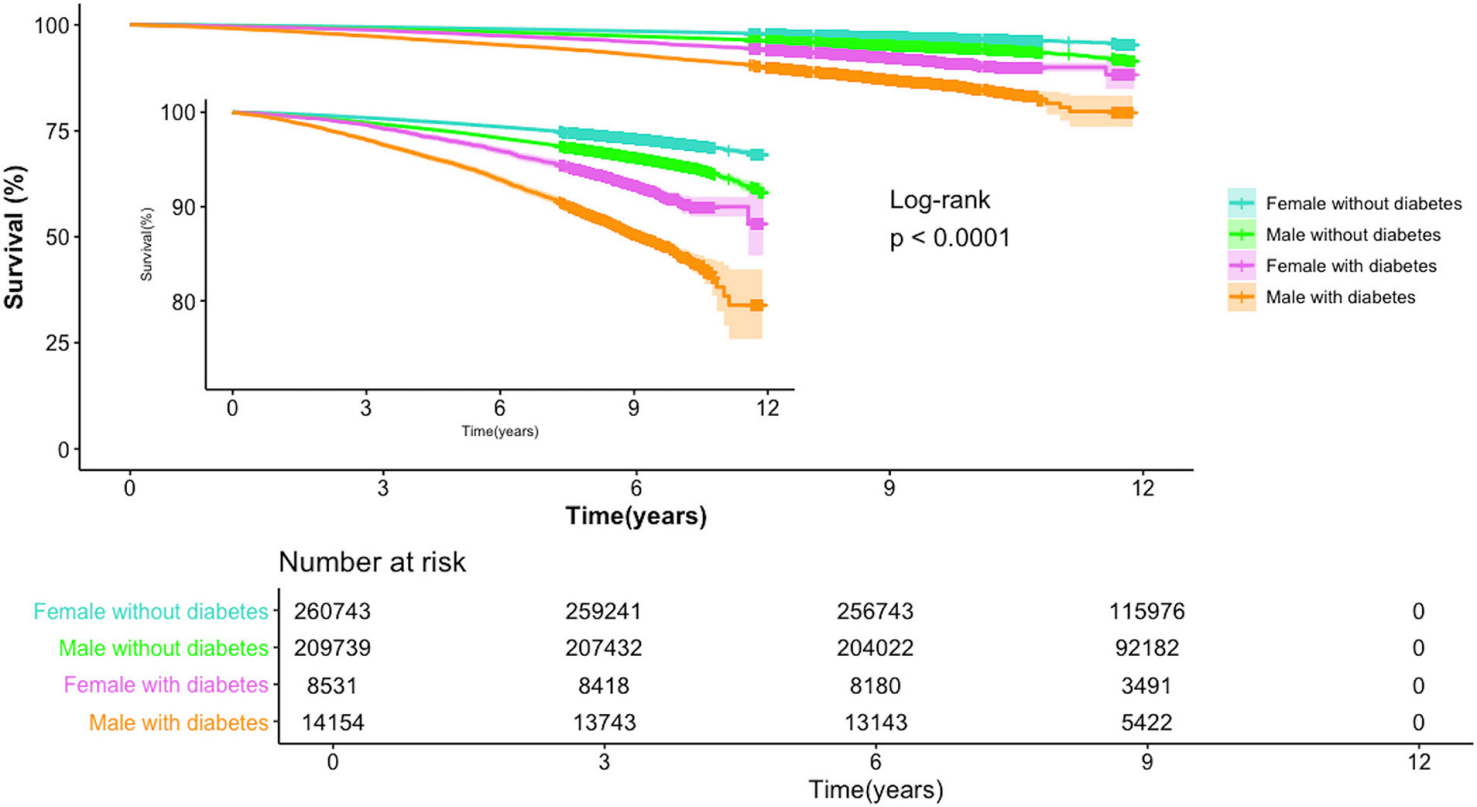
The risk of all-cause mortality according to sex and presence of diabetes.

**Figure 2:**
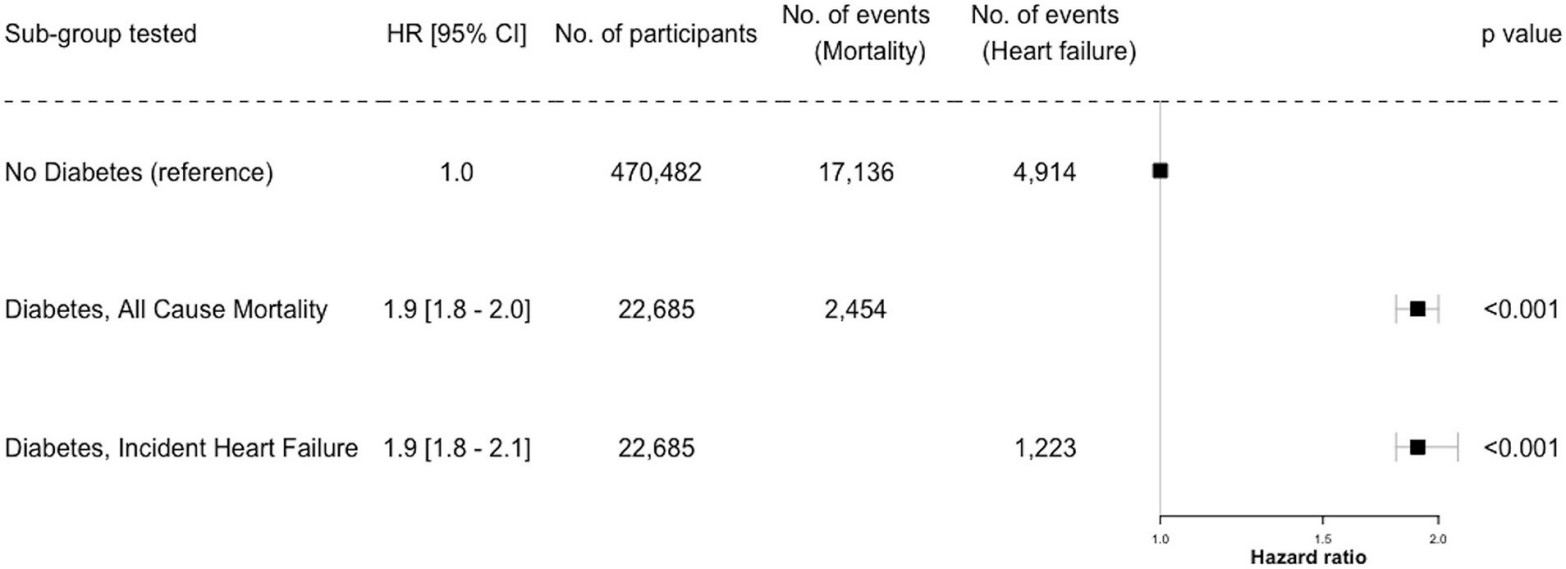
Risk of All-Cause Mortality and Incident Heart Failure in Diabetes. HR, hazard ratio (95% confidence interval shown).

A univariate analysis showed that those with diabetes have had a two-fold higher risk of mortality compared to individuals without diabetes. Men were found to have a lower survival probability compared to women in both groups—with and without diabetes. These results are demonstrated in Figure 1. In the multivariate analysis—diabetes was associated with almost double the mortality risk compared to those without diabetes (Figure 2). Further analysis showed that the excess risk of mortality in patients with diabetes is slightly higher in women compared to men (Supplementary Table 1).

### Diabetes and Heart Failure

**Figure 3:**
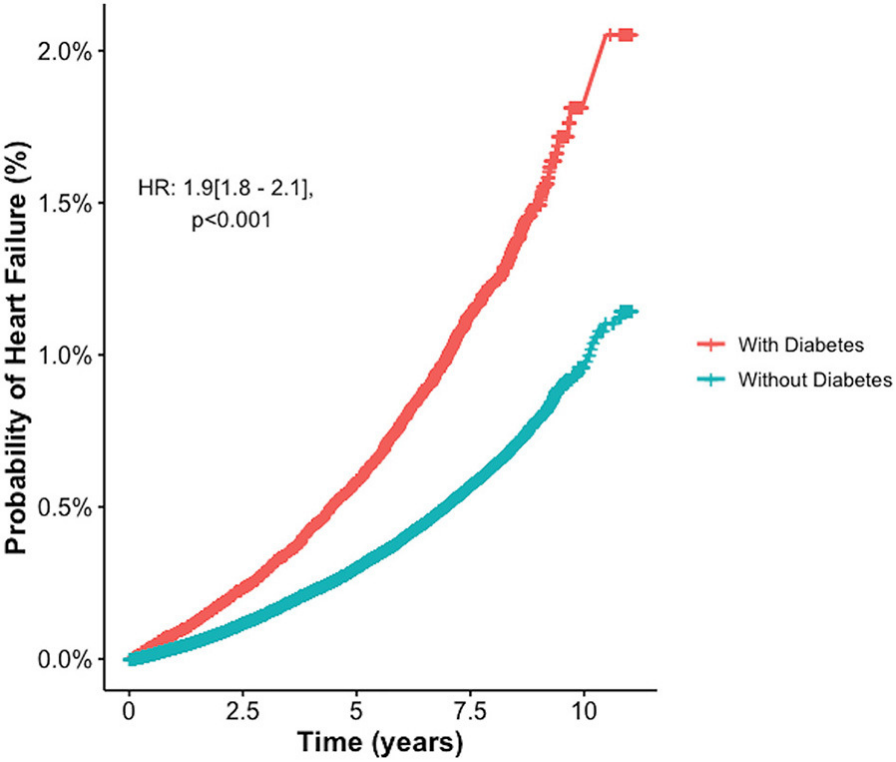
Increased probability of incident Heart Failure in Diabetes: a multivariate analysis. HR, hazard ratio.

Similarly, examining the relationship between diabetes status and incident heart failure, a multivariate analysis showed those with diabetes were at almost double the risk of heart failure compared to those without (Figures 2, 3).

### Sex Differences in Risk of Heart Failure

**Figure 4:**
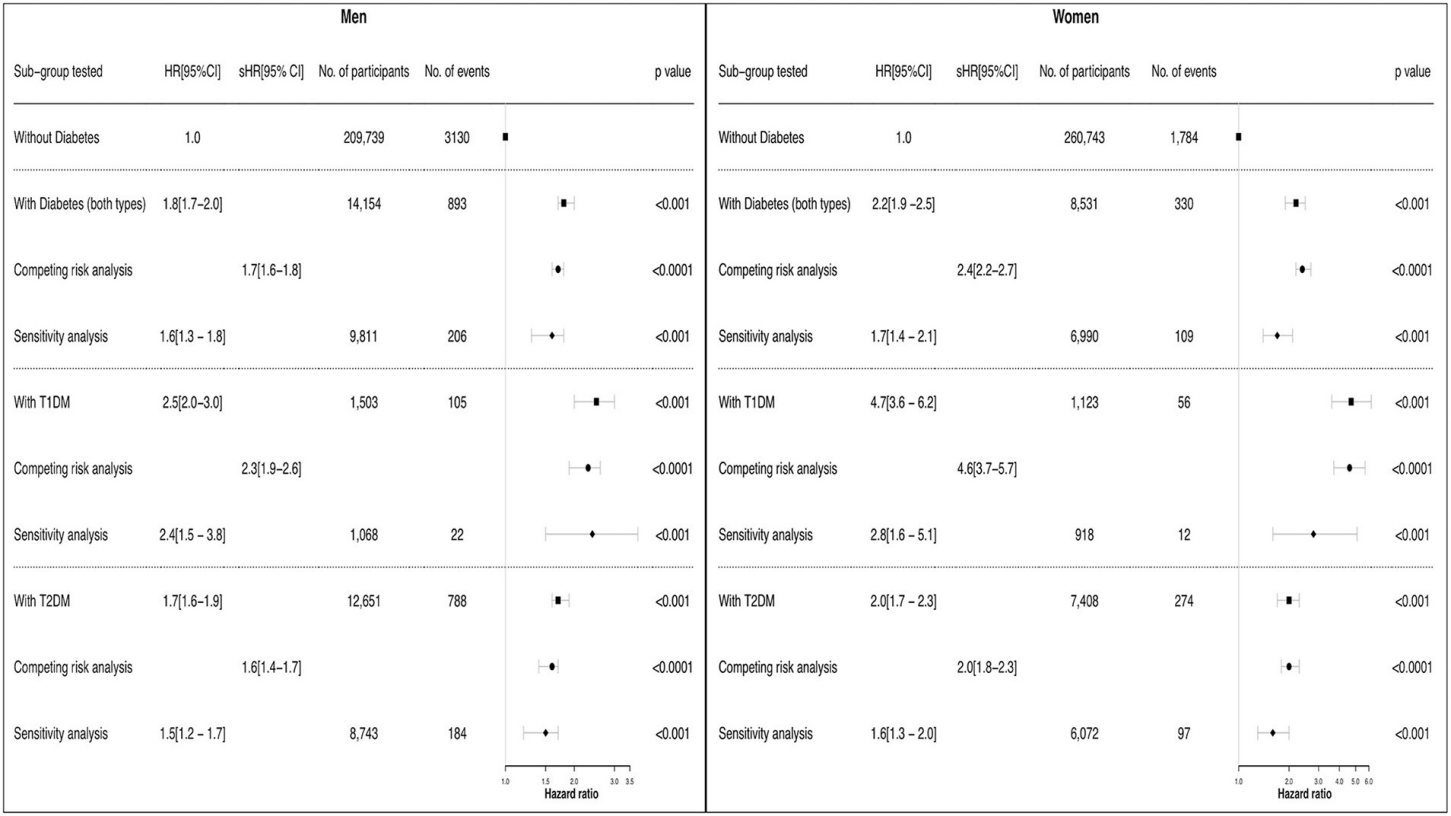
Association between Diabetes, Gender and Incident of Heart Failure – multivariate, competitive risk and sensitivity analysis. Forest plot demonstrating risk of HF between men and women for each subset of participants with diabetes. The multivariate cox models were adjusted for age, ethnicity, hypertension, hypercholesterolaemia, smoking, BMI, alcohol status with coronary artery disease stratified. Interaction term between sex and heart failure is significant in the T1DM group (*p* = 0.0001) and for the overall diabetes group (*p* = 0.007). Interaction term for sex and heart failure in T2DM is *p* = 0.1. Competing risk confirms the trend seen in the multivariate analysis, and indicates that the increased risk in women especially with T1DM is significant enough to be above all-cause mortality. T1DM, type 1 diabetes; T2DM, type 2 diabetes; HR, hazard ratio; sHR, sub-distribution hazard ratio.

Figure 4 demonstrates the risk of heart failure for men and women with and without diabetes in the UK Biobank population, with models adjusted for our pre-defined confounding variables. The population was stratified into sex, and the association with incident heart failure was examined for type 1 diabetes, type 2 diabetes and all individuals with diabetes. As shown, there was an increased risk of heart failure with diabetes in both men and women, with absolute risk of events increased in men. Relative risk estimates were higher for type 1 diabetes than type 2 diabetes and, interestingly, the effect of diabetes was greater for women than for men. For women, the risk of heart failure associated with diabetes from the multivariate model, type 1 and type 2 combined, was 22% higher than for men, with hazard ratios of 2.2 (95% CI: 1.9–2.5) and 1.8 (1.7–2.0) respectively (*p*-value for interaction = 0.007). When stratified into type 1 and type 2 diabetes the risk of heart failure associated with type 1 diabetes was 88% higher in women compared to men (hazard ratios 4.7 (3.6–6.2) vs. 2.5 (2.0–3.0), interaction *p* = 0.0001), while the risk of heart failure for type 2 diabetes was 17% higher in women compared to men (hazard ratios 2.0 (1.7–2.3) vs. 1.7 (1.6–1.9), interaction *p* = 0.10). Overall, findings were similar in the competing risk analyses indicating that the increased risk associated with type 1 diabetes in women remains even after accounting for the effect of all-cause mortality as a competing risk. The bootstrap analysis of the difference between coefficient of men and women in different sub-groups confirms the interaction term analysis.

A sensitivity analysis excluding individuals with coronary artery disease showed that the hazard ratios were still higher for women with diabetes than men, however, the significant interaction effect seen in the other analysis was not demonstrated (Figure 4). The reason for this was thought to be due to the lower number of events observed once those with coronary disease were removed. Further clarification was sought using mediation analysis which showed coronary disease may not have any mediatory effect in men with diabetes but in women with diabetes: 20% (19.1–20.9) mediatory effect is seen (see Supplementary Table 2).

## Discussion

Men with diabetes have an increased absolute risk of heart failure events. However, the main finding of the present paper is that in women, the relative risk of suffering from heart failure for diabetes compared to those without diabetes is higher than in men, despite adjusting and stratifying for confounding variables. Although coronary disease has a minimal mediatory effect, it does not explain the majority of the excess risk of heart failure seen in women. Interestingly, the increased risk is particularly prominent in women with type 1 diabetes. The competing risk analysis in women with type 1 diabetes highlighted that the increased risk of heart failure remains after accounting for the effect of all-cause mortality as a competing risk. For type 2 diabetes the multivariate cox analysis shows the same trend where women are at increased risk of suffering from heart failure compared to men, however, the interaction between sex and heart failure was not statistically significant.

Although heart failure is the main focus of this study, a multivariate analysis showed that the excess risk of mortality in patients with diabetes is higher in women compared to men (Supplementary Table 1). This finding is supported by previous large cohort studies, which had found that women with diabetes had increased rates of cardiovascular and renal events as causes of death (18, 19).

This study is the largest to investigate the potential factors contributing to sex dependent difference in risk of heart failure associated with diabetes. The findings are in agreement with the results from a large meta-analysis consisting of 12 million people which was showing that having diabetes increased the risk of heart failure in women more than in men, an effect which was strongest when looking at type 1 diabetes (11). However, in the present study, we could take potential confounding factors into consideration, thereby significantly strengthening the observations. Our findings therefore suggest that the increased risk of heart failure associated with diabetes in women compared to men is not fully explained by confounding factors, but is likely a biological difference in the effect of diabetes on cardiac function in women compared to men, most notably in those with type 1 diabetes.

A widely suggested mechanism is that the increased risk in heart failure in women is secondary to the increased risk of coronary heart disease established in other studies (20, 21). The mediation analysis shows that coronary disease does have a mediatory effect on the outcome of heart failure in women with diabetes, but cannot fully account for the excess risk seen through multivariate cox regression analysis where coronary disease and other confounding variables are accounted for.

In summary, these findings may suggest that diabetes is a discrete cause of heart failure and affects women more than men, particularly in type 1 diabetes.

Sex based differences in cardiac physiology in the healthy population have been observed (22). After puberty, it is noted that male hearts undergo greater hypertrophy than women (23). In an otherwise healthy population, aging leads to an increase in septal thickness in both men and women, but the left ventricular diameter is noted to increase only in men (24) and results in loss of myocardial mass due to loss of myocytes. This loss is thought to result in compensatory hypertrophy of remaining myocytes in men, whereas myocyte mass and size are preserved in healthy women (25). These cellular changes may result in women having better diastolic function and preserved systolic function compared to men (24). Furthermore, in a healthy population, the mechanisms of cardiac adaption to exercise have been shown to be inherently different in male and female hearts despite the end result being an increase in cardiac output (26, 27). These differences in cardiac physiology and function are mostly lost in post-menopausal women (22). This would suggest that sex hormones play a role in the development and maintenance of normal cardiac function in healthy women, which is possibly reliant on a greater degree of elasticity (diastolic function). Diastolic dysfunction is a hallmark of diabetic cardiomyopathy (28). Therefore, it is possible that the benefits inferred by sex-based differences in healthy women are opposed or reduced in women with diabetes. The loss of diastolic function has a greater detrimental effect on female hearts compared to men. This may be a potential explanation for the findings of the prior metanalysis and this study.

It has also been suggested that women may be worse affected than men because they are traditionally noted to have worse glycaemic and cardiac risk factor control (29, 30). However, in this cohort the HbA1c levels are well-matched between men and women. Additionally, the traditional cardiac risk factors were adjusted for in the regression model. Therefore, the findings of this study do not support the theory that additional risk women with diabetes face is attributed to poorer glycaemic control and risk factor management.

### Interaction Between Sex, Diabetes and Heart Failure

The interaction between sex and diabetes on risk of heart failure for type 1 diabetes is statistically significant, unlike in type 2 diabetes. A recent study supports these findings and has also demonstrated certain imaging markers that are prognostic indicators of outcome for women with type 1 diabetes compared to men (31). This suggests that there are mechanisms to investigate that may correlate to the epidemiological findings of this study. Although both types of diabetes are characterized by hyperglycaemia, they are very different conditions in terms of pathophysiology and effect on cell metabolism (32), which could account for the difference seen between the two groups in this study. In addition, those with type 1 diabetes have often been diagnosed at a younger age and therefore have had a longer duration of disease which may also be responsible for the difference seen between the risk of heart failure in type 1 diabetes compared to type 2 diabetes. There has been some suggestion that insulin therapy itself may cause cardiac dysfunction and this could contribute to the excess risk of heart failure amongst those with diabetes (33, 34). This could potentially be an explanation for the increased risk in type 1 diabetes compared to type 2 diabetes. However, current literature in support of this theory is limited to animal models and explored in relation to insulin excess generated in the metabolic intolerance state in type 2 diabetes.

There is also some evidence suggesting that there are sex specific differences in telomerase activity in the heart and other molecular mechanisms, which may lead to the difference in disease expression amongst men and women with diabetes (35–37).

Overall, the evidence from this large study suggests that an independent process (diabetic cardiomyopathy) may be a potential mechanism that leads to the excess risk of heart failure in women with diabetes. Our recent study has shown that there are structural and functional changes associated with those with diabetes independent of coronary artery disease in the UK Biobank population (38). This study was performed using the CMR images from the first 5,000 participants that were scanned as part of the imaging study.

## Limitations

One of the limitations in this study, however, is that the HbA1c is a measurement taken at enrolment for the participants in UK Biobank and does not necessarily reflect long term glycaemic control. The generalizability of the findings of this study to the general population may also be a limitation. Participants in the UK Biobank are volunteers who are motivated and actively participated in this study, and therefore are generally recognized as having increased health awareness, resulting in the overall cohort being “healthier” than the general population. However, if excess risk in people with diabetes and women with diabetes can be detected in this population, then it could be surmised that this excess risk is even more likely to be present in the general population. Finally, the type of heart failure that participants are diagnosed with is not distinguished, therefore we cannot separate the outcomes of HF with reduced ejection fraction and HF with preserved ejection fraction in this study.

## Conclusions

Both men and women with diabetes are more likely to develop heart failure compared to their non-diabetic counterparts, however for women this excess risk is significantly greater than for men. This finding is more significant for type 1 diabetes than type 2 diabetes. The increased relative risk for women cannot be explained solely by factors such as increased prevalence of coronary artery disease and other cardiac risk factors. Therefore, diabetic cardiomyopathy, myocardial dysfunction related to diabetes, is a potential contributor, which affects women more than men. In order to identify this condition and develop sex specific treatment strategies, further research is needed to first establish the cardiac phenotype of diabetic cardiomyopathy and the sex differences within this phenotype. Defining this condition will allow for screening and treatment strategies to be developed and targeted.

## Data Availability Statement

Publicly available datasets were analyzed in this study. This data can be found at: http://www.ukbiobank.ac.uk/register-apply/.

## Author Contributions

SC is the first author and was involved in the conceptualization, data collation, data analysis, and manuscript preparation. MJ, NA, JC, KL, and PM have all contributed equally to this work and involved in data analysis and manuscript preparation. SP is the senior author and has supervised all aspects of the study and contributed to the manuscript preparation. All authors contributed to the article and approved the submitted version.

## Conflict of Interest

The authors declare that the research was conducted in the absence of any commercial or financial relationships that could be construed as a potential conflict of interest.

## Appendix

**Appendix A:**

**Variable in study**	**Definition within UK Biobank database**
Ethnicity	Derived from self-reported questionnaire participants answer at first assessment.
Smoking history	Derived from self-reported questionnaire participants answer at first assessment where participants answered if they were a current, previous, never smoked, or prefer not to answer.
Alcohol history	Derived from self-reported questionnaire participants answer at first assessment where participants answered if they were a current, previous, never smoked, or prefer not to answer.
Hypertension	Derived from self-reported questionnaire given to UK Biobank participants and HES data. This was supplemented with data on those participants taking anti-hypertensive medications.
Coronary disease	Derived from self-reported questionnaire given to UK Biobank participants and HES data including ICD 10 codes 120 – 125. In addition, any participants with hospital admission for coronary intervention (percutaneous or surgical bypass grafting) were also recorded to have coronary disease.
Hypercholesterolaemia	Derived from self-reported questionnaire given to UK Biobank participants. This was supplemented with data on those participants taking statin medication.
Heart Failure	Derived from self-reported questionnaire given to UK Biobank participants and HES data including ICD code 150.
Diabetic medication	Derived from self-reported medication, supplemented with data on patients self-reported to be on insulin or those started on insulin within a year of diabetes diagnosis.
