# Supplementary material for: Women With Diabetes Are at Increased Relative Risk of Heart Failure Compared to Men: Insights From UK Biobank
Source: Front Cardiovasc Med. 2021 Apr 6;8:658726. doi: 10.3389/fcvm.2021.658726 (PMC8057521; doi:10.3389/fcvm.2021.658726)
Supplement: Supplementary file 1 [file Data_Sheet_1.docx]

**SUPPLEMENTARY TABLES**

**Supplementary Table 1** – Hazard ratios for mortality according to sex and type of diabetes

| Mortality | Men | 95% Confidence interval | P value | Women | 95% Confidence interval | P value |
| --- | --- | --- | --- | --- | --- | --- |
| Overall Diabetes vs non-diabetes | 1.8 | 1.7 – 2.0 | <0.001 | 1.9 | 1. 7– 2.0 | <0.001 |
| T1DM | 3.3 | 2.9 – 3.7 | <0.001 | 3.9 | 3.2 – 4.7 | <0.001 |
| T2DM | 1.8 | 1.7 – 1.9 | <0.001 | 1.8 | 1.6 – 2.0 | <0.001 |

**Supplementary Table 2** - Mediation analysis results for heart failure according to sex with coronary disease as mediator and type of diabetes as the exposure.

| Proportion mediated | | | | | | |
| --- | --- | --- | --- | --- | --- | --- |
|  | Men | 95% Confidence interval | P value | Women | 95% Confidence interval | P value |
| Diabetes (both types) | -0.033 | -0.038 to -0.027 | <0.0001 | 0.200 | 0.191 to 0.209 | <0.0001 |
| T1DM | 0.012 | -0.002 to 0.025 | 0.093 | 0.263 | 0.244 to 0.282 | <0.0001 |
| T2DM | -0.041 | -0.047 to -0.034 | <0.0001 | 0.197 | 0.188 to 0.206 | <0.0001 |
| Total effect HR | | | | | | |
| Diabetes (both types) | 1.84 | 1.69 to 2.00 | <0.0001 | 2.61 | 2.29 to 2.97 | <0.0001 |
| T1DM | 2.63 | 2.14 to 3.24 | <0.0001 | 6.20 | 4.70 to 8.19 | <0.0001 |
| T2DM | 1.77 | 1.62 to 1.93 | <0.0001 | 2.30 | 2.00 to 2.65 | <0.0001 |
| Total Indirect effect HR | | | | | | |
| Diabetes (both types) | 0.99 | 0.97 to 1.00 | 0.02 | 1.14 | 1.11 to 1.17 | <0.0001 |
| T1DM | 1.01 | 0.97 to 1.05 | 0.698 | 1.29 | 1.20 to 1.38 | <0.0001 |
| T2DM | 0.98 | 0.97 to 1.00 | 0.01 | 1.13 | 1.10 to 1.15 | <0.0001 |
| Total direct effect HR | | | | | | |
| Diabetes (both types) | 1.87 | 1.72 to 2.03 | <0.0001 | 2.28 | 2.00 to 2.61 | <0.0001 |
| T1DM | 2.62 | 2.13 to 3.21 | <0.0001 | 4.83 | 3.68 to 6.35 | <0.0001 |
| T2DM | 1.80 | 1.65 to 1.96 | <0.0001 | 2.04 | 1.77 to 2.35 | <0.0001 |

**Supplementary table 2 footnote**: HR: Hazard ratio.
